# Calcineurin Is Required for Pseudohyphal Growth, Virulence, and Drug Resistance in *Candida lusitaniae*

**DOI:** 10.1371/journal.pone.0044192

**Published:** 2012-08-31

**Authors:** Jing Zhang, Fitz Gerald S. Silao, Ursela G. Bigol, Alice Alma C. Bungay, Marilou G. Nicolas, Joseph Heitman, Ying-Lien Chen

**Affiliations:** 1 Department of Molecular Genetics and Microbiology, Duke University, Durham, North Carolina, United States of America; 2 Department of Chemistry, Duke University, Durham, North Carolina, United States of America; 3 Department of Microbiology and Parasitology, University of Perpetual Help – Dr. Jose G. Tamayo Medical University, Biñan, Laguna, Philippines; 4 Environment and Biotechnology Division, Department of Science and Technology, Bicutan, Philippines; 5 National Institutes of Health-Philippines, University of the Philippines, Manila, Philippines; Yonsei University, Republic of Korea

## Abstract

*Candida lusitaniae* is an emerging fungal pathogen that infects immunocompromised patients including HIV/AIDS, cancer, and neonatal pediatric patients. Though less prevalent than other *Candida* species, *C. lusitaniae* is unique in its ability to develop resistance to amphotericin B. We investigated the role of the calcium-activated protein phosphatase calcineurin in several virulence attributes of *C. lusitaniae* including pseudohyphal growth, serum survival, and growth at 37°C. We found that calcineurin and Crz1, a *C. albicans* Crz1 homolog acting as a downstream target of calcineurin, are required for *C. lusitaniae* pseudohyphal growth, a process for which the underlying mechanism remains largely unknown in *C. lusitaniae* but hyphal growth is fundamental to *C. albicans* virulence. We demonstrate that calcineurin is required for cell wall integrity, ER stress response, optimal growth in serum, virulence in a murine systemic infection model, and antifungal drug tolerance in *C. lusitaniae*. To further examine the potential of targeting the calcineurin signaling cascade for antifungal drug development, we examined the activity of a calcineurin inhibitor FK506 in combination with caspofungin against echinocandin resistant *C. lusitaniae* clinical isolates. Broth microdilution and drug disk diffusion assays demonstrate that FK506 has synergistic fungicidal activity with caspofungin against echinocandin resistant isolates. Our findings reveal that pseudohyphal growth is controlled by the calcineurin signaling cascade, and highlight the potential use of calcineurin inhibitors and caspofungin for emerging drug-resistant *C. lusitaniae* infections.

## Introduction

*Candida lusitaniae* (teleomorph *Clavispora lusitaniae*) is a sexual, haploid, emerging yeast pathogen that infects immunocompromised patients with co-morbid conditions such as cancer and HIV/AIDS [1], [2], [3], [4], [5]. Since it was first documented in 1979 in the context of an opportunistic infection in a patient with acute leukemia [6], *C. lusitaniae* has been recovered from various sites in the human body including urine, bronchoalveolar lavage fluid, blood, and peritoneal fluid, and from the kidney, vagina, and skin [7], [8], [9]. Though less prevalent than other *Candida* species in causing only 0.6 ∼ 2.0% of all cases of candidemia [10], *C. lusitaniae* exhibits a unique predilection to readily develop resistance to the antifungal agents amphotericin B, flucyotsine, and fluconazole, which poses a major obstacle in its treatment [3], [4], [11], [12]. Amphotericin B resistance is controlled by the ergosterol biosynthetic gene *ERG6* and possibly also *ERG3* [13], but mechanisms that control flucytosine and fluconazole resistance remain unclear in *C. lusitaniae*. More recently, 5.4% of non-neonatal pediatric cases of candidemia were found to be attributable to *C. lusitaniae* [14].

Furthermore, clinical isolates of *C. lusitaniae* resistant to the echinocandins due to a missense mutation (S645F) in the Fks1 protein have been reported [8]. Past studies have documented that the protein phosphatase calcineurin and its downstream target Crz1 play important roles in virulence and drug tolerance of *C. albicans*, *C. dubliniensis*, and *C. glabrata* [15], [16], [17], [18]. Calcineurin, which is comprised of a catalytic A (Cna1) and a regulatory B (Cnb1) subunit, dephosphorylates several proteins including the transcription factor Crz1 in fungi and the nuclear factor of activated T cells (NFAT) in mammals when stimulated by Ca^2+^-calmodulin. The calcineurin inhibitor FK506 has previously been shown to exhibit synergistic fungicidal activity with caspofungin against a *C. dubliniensis* echinocandin-resistant strain [19], suggesting that combination antimicrobial treatment with FK506 and echinocandins is promising in the development of novel therapies against emerging *C. lusitaniae* infections.

Calcineurin is involved in hyphal growth of *C. dubliniensis* [15], *Aspergillus fumigatus* [20], and *Cryptococcus neoformans* [21], [22]. However, its roles in *C. albicans* hyphal growth remain ambiguous. The ability to switch to hyphal growth is central to fungal virulence and amongst the *Candida* species, is unique to *C. albicans*, *C. dubliniensis,* and *C. tropicalis*. *C. lusitaniae* is able to form pseudohyphae although its ability to form true hyphae as well as the role of calcineurin signaling in these processes has so far remained elusive. Genes regulating pseudohyphal growth of *C. lusitaniae* include *SLN1*, which encodes a class VI histidine kinase receptor [23], and *SSK1*, encoding a downstream response regulator, are both required for the early steps of pseudohyphal growth [24]. Interestingly, the dimorphic transition between yeast and filamentous growth has been implicated in *C. lusitaniae* amphotericin B (AmB) resistance. Miller et al. found that phenotypic switching of an AmB-resistant *C. lusitaniae* strain on CuSO_4_ media resulted in light brown, dark brown, or white colonies. Filamentation (pseudohyphae) was seen only in dark brown colonies that had AmB minimum inhibitory values (MIC) intermediate between light brown and high-AmB resistant white colonies [25].

The roles of the calcineurin signaling cascade in controlling serum survival and growth at 37°C have been investigated. Previous studies have shown that calcineurin is required for serum survival in *C. albicans* [26] and growth at 37°C or above in *C. glabrata* [16].

Although caspofungin is often used as a first-line therapy to treat *C. lusitaniae* infections in patients pre-exposed to azole drugs, clinical *C. lusitaniae* isolates resistant to the echinocandins have been reported [8]. These isolates have a missense mutation in the *FKS1* gene, which encodes β-1,3-glucan synthase, the target of the echinocandin drugs [27]. Previous research has shown that calcineurin is required for echinocandin and azole drug tolerance in *C. albicans*, *C. dubliniensis*, and *C. glabrata* [15], [16], [17]. Furthermore, calcineurin inhibitor FK506 exhibits synergistic fungicidal activity with caspofungin against a *C. dubliniensis* echinocandin-resistant strain [19].

The emergence of *C. lusitaniae* as a serious cause of systemic fungal infections, as well as this pathogen’s antifungal resistance patterns, suggests a need to further investigate improved treatment options. In this study we explore the role of calcineurin and Crz1 in pseudohyphal development, virulence properties, and drug tolerance of *C. lusitaniae*. We demonstrate that calcineurin and/or Crz1 are required for *C. lusitaniae* pseudohyphal growth, optimal growth in serum, and Ca^2+^ homeostasis. Furthermore, we show that *C. lusitaniae* calcineurin and Crz1 contribute to virulence in a murine systemic infection model, and that a calcineurin inhibitor exhibits synergistic antifungal activity with caspofungin against two clinical echinocandin-resistant *C. lusitaniae* isolates *in vitro*.

## Results and Discussion

### Calcineurin is Essential for Cell Wall Integrity and ER Stress in *C. lusitaniae*

The *C. lusitaniae* orthologs of *C. albicans* and *S. cerevisiae CNB1* and the calcineurin target *CRZ1* genes were identified by reciprocal BLAST searches between the two species and in both cases identified a reciprocal best BLAST hit ortholog as the *C. lusitaniae CNB1* (CLUG_00707.1) and *CRZ1* (CLUG_04763.1) genes [28]. *C. lusitaniae* Cnb1 shares 73% and 70% (Figure S1A), while Crz1 shares 32% and 22% identity (Figure S2A) over the full protein lengths with their corresponding *C. albicans* and *S. cerevisiae* orthologs, respectively. The *C. lusitaniae* calcineurin B (Cnb1) protein has four EF-hand Ca^2+^ binding motifs (Figure S1B), while Crz1 shares two C_2_H_2_ zinc finger motifs with the respective orthologs in *C. albicans* and *S. cerevisiae* (Figure S2B). Two independent *cnb1* and *crz1* mutants were generated using the *SAT1* marker and confirmed by PCR and Southern blot analysis.

**Figure 1:**
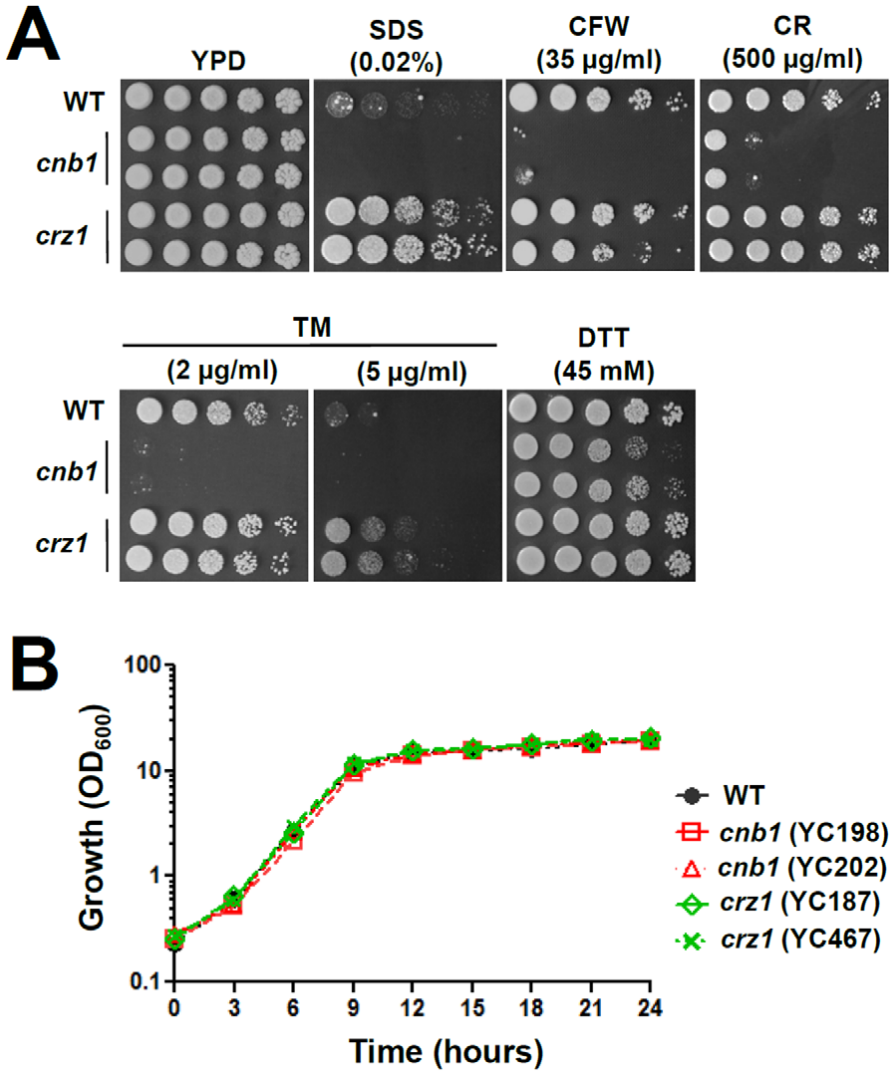
Calcineurin mutation confers cell wall integrity defects in *C. lusitaniae*. (**A**) Calcineurin mutants are sensitive to cell wall integrity-damaging agents and ER stress chemicals. Cells were grown overnight in YPD at 30°C, 5-fold serially diluted, and spotted onto YPD medium containing sodium dodecyl sulfate (SDS), calcofluor white (CFW), congo red (CR), tunicamycin (TM), or dithiothreitol (DTT) and incubated at 30°C for 48 h. (**B**) The growth kinetics of *C. lusitaniae* wild-type and mutant strains at 30°C. Cells were grown overnight at 30°C, washed twice with dH_2_O, diluted to 0.2 OD_600_/ml in fresh liquid YPD medium, and incubated at 30°C with shaking at 250 rpm. The OD_600_ of cultures was measured at 0, 3, 6, 9, 12, 15, 18, 21, and 24 h. The experiments were performed in triplicate, and data was plotted using Prism 5.03. Strains tested were wild-type (ATCC42720), *cnb1* mutants (YC198 and YC202), and *crz1* mutants (YC187 and YC467).

Because calcineurin plays a general role in controlling cell wall integrity in most fungal pathogens, we first characterized the potential requirement of calcineurin in response to cell membrane/wall disturbing agents, e.g. sodium dodecyl sulfate (SDS), calcofluor white (CFW), and congo red (CR). SDS compromises cell membrane integrity [29], CFW destabilizes chitin polymerization [30], and CR intercalates between glucan polymers [31]. The *cnb1*, but not *crz1*, mutants are sensitive to SDS, CFW, and CR (Figure 1A). Interestingly, in contrast to the SDS sensitivity of *C. albicans* and *C. dubliniensis crz1/crz1* mutants that is intermediate between wild-type and calcineurin mutants [15], *C. lusitaniae crz1* mutants exhibit resistance to SDS (Figure 1A), suggesting that Crz1 negatively regulates cell membrane integrity and an unknown calcineurin independent factor controls Crz1 in response to SDS (Figure 8). Similar to SDS, *C. lusitaniae cnb1* mutants show sensitivity to the ER stress inducer tunicamycin, while *crz1* mutants exhibit modest resistance (Figure 1A). In addition to inducing ER stress, tunicamycin also blocks N-glycosylation of cell wall proteins. Therefore, the ER sensitivity of calcineurin mutants might also be attributable to defective cell wall integrity induced by tunicamycin. The observed sensitivity to chemical compounds does not result from slower growth because wild-type and calcineurin signaling mutants exhibit similar growth kinetics in YPD at 30°C (Figure 1B). In addition, we tested if *C. lusitaniae* calcineurin plays a role in controlling thermotolerance, as seen in *C. glabrata* [16]. We found that most (11/12; 91.7%) *C. lusitaniae* isolates do not exhibit thermal sensitivity in the presence of FK506 or cyclosporin A (Figure S3). Interestingly, one out of 12 (8.3%) *C. lusitaniae* strains tested exhibited sensitivity at 37°C (Figure S3), which is similar but less than the 16% (3/19) of *C. glabrata* strains tested showed that temperature sensitivity in the presence of FK506 or CsA [16].

### Calcineurin and Crz1 Control Pseudohyphal Growth in *C. lusitaniae*

**Figure 2:**
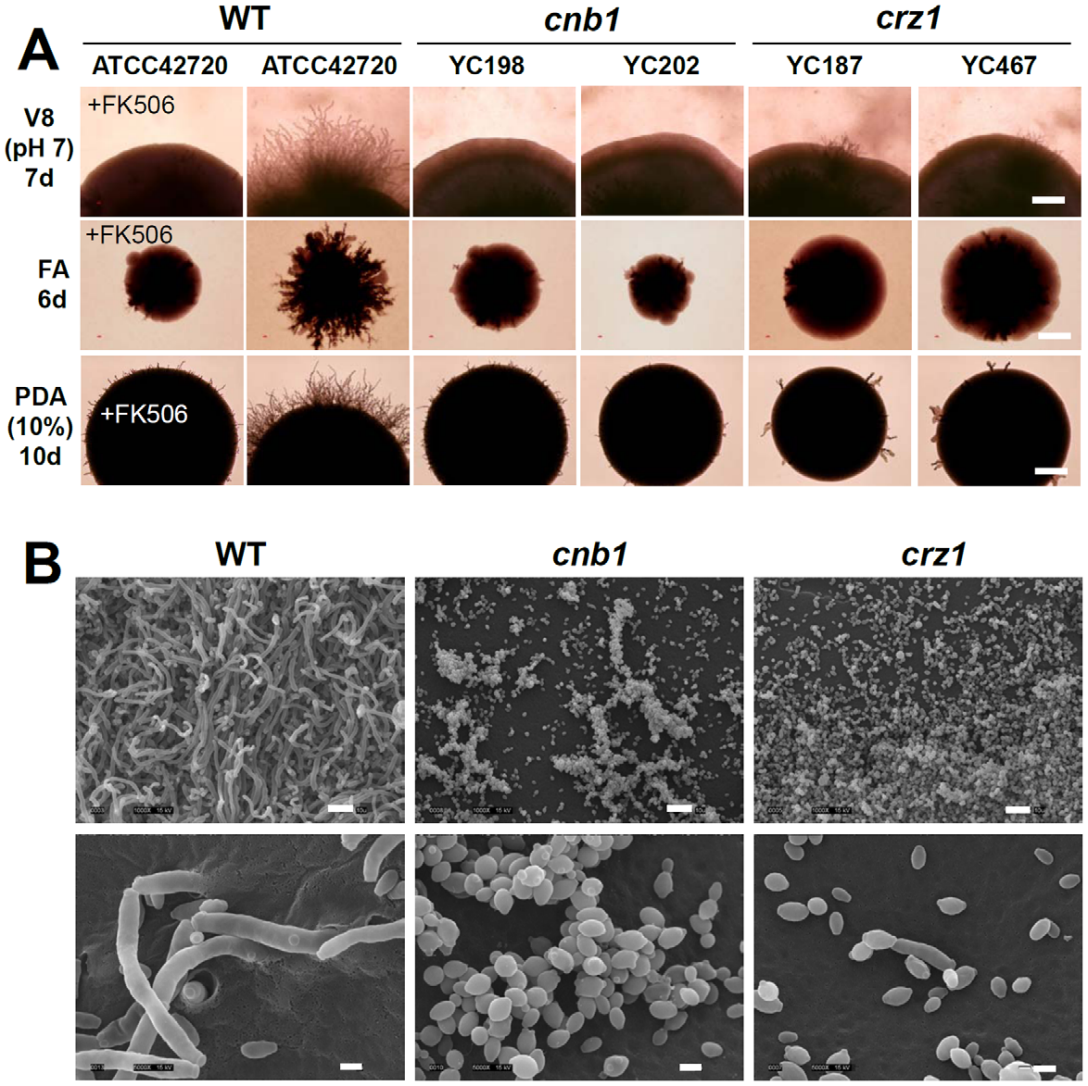
Calcineurin and Crz1 control colony pseudohyphal growth of *C. lusitaniae*. (**A**) Cells were grown overnight and washed twice with dH_2_O. Cells were diluted to 500 cells/ml. One hundred microliters containing ∼50 cells were spread on a variety of filament-inducing media lacking or containing FK506 (1 µg/ml), and incubated at 37°C for the number of days indicated. FA, Filament Agar; PDA, Potato Dextrose Agar. The experiments were repeated at least three times and one representative image is shown. Scale bar = 0.5 mm. (**B**) Scanning electron microscopy (SEM) images of *C. lusitaniae* on filament-inducing media. Cells grown on V8 (pH = 7) media for 7 days at 37°C were processed for SEM, and imaged (see Materials and Methods). Scale bars for upper panel (1000x) and lower panel (5000x) images represent 10 µm and 2 µm, respectively. WT (ATCC42720), *cnb1* mutant (YC198), and *crz1* mutant (YC187).

*C. lusitaniae* can switch from yeast to pseudohyphal growth when cultured on a solid medium such as yeast carbon base, V8, or potato dextrose agar (PDA). Information regarding the mechanisms that regulate pseudohyphal growth in *C. lusitaniae* is limited. Previous studies have reported that *C. lusitaniae* HOG pathway components Ssk1, Ssk2, Pbs2, Hog1, and Sho1 play roles in pseudohyphal development [24], [32], [33]. Although *C. lusitaniae* Cls12, a Ste12 homolog of *S. cerevisiae*, is required for mating, it is dispensable for pseudohyphal growth [34]. The dimorphic transition (yeast to pseudohyphae) in *C. lusitaniae* has been associated with dark brown colony formation when exposed to copper sulfate [25]. Interestingly, true hyphae have thus far not been reported in *C. lusitaniae*. Here, we demonstrate that pseudohyphal growth is controlled by calcineurin and Crz1 in *C. lusitaniae* (Figure 2 and Figure 8). Calcineurin and *crz1* mutants exhibit pseudohyphal growth defects on filament-inducing V8 (pH = 7), filament agar (FA; no added nitrogen source), and PDA (10%) solid media (Figure 2A). In the presence of the calcineurin inhibitor FK506, the *C. lusitaniae* wild-type exhibits defective pseudohyphal growth (Figure 2A), supporting the phenotypes of genetically disrupting calcineurin. In scanning electron microscopy analysis, we found that the *C. lusitaniae* type strain ATCC42720 is able to invade the solid agar and forms pseudohyphae (Figures 2B and S4), while *cnb1* and *crz1* mutants only exhibit yeast growth (Figure 2B). Further studies to identify calcineurin and Crz1 downstream targets controlling *C. lusitaniae* pseudohyphal growth would be interesting, as would comparisons with the pseudohyphal and hyphal growth machinery in *C. albicans*.

### Calcineurin is Required for Optimal *C. lusitaniae* Growth in Serum

**Figure 3:**
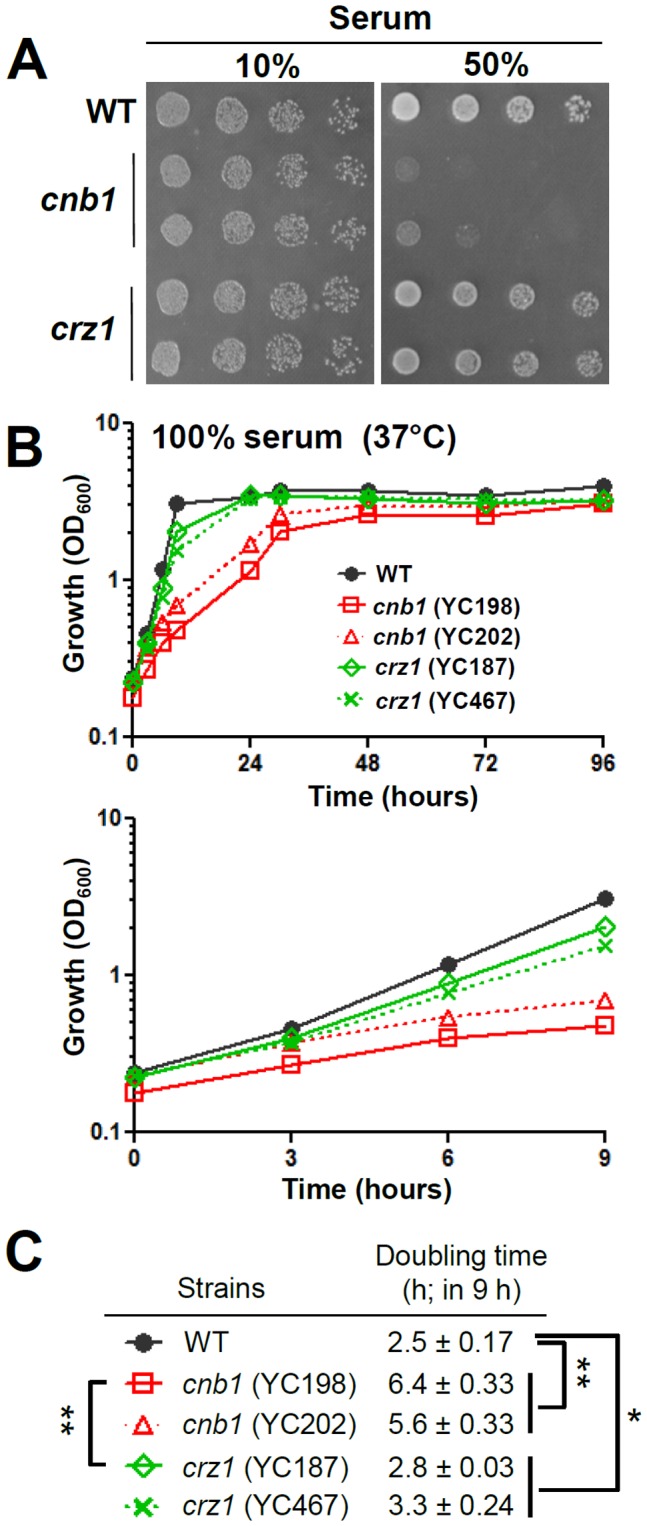
Optimal growth in serum is controlled by calcineurin in *C. lusitaniae*. (**A**) Cells were grown overnight in YPD at 30°C, 5-fold serially diluted, and spotted onto agar water medium containing 10% or 50% fetal bovine serum, and incubated at 37°C for 48 h. (**B**) The growth kinetics of *C. lusitaniae* wild-type and mutant strains on 100% serum at 37°C. Cells were grown overnight at 30°C, washed twice with dH_2_O, diluted to 0.2 OD_600_/ml in 100% serum, and incubated at 37°C with shaking at 250 rpm. The OD_600_ of cultures was measured at 0, 3, 6, 9, 24, 30, 48, 72, and 96 h (upper panel). The lower panel shows the growth kinetics between 0 and 9 h extracted from the upper panel. The experiments were performed in triplicate, and data was plotted using Prism 5.03. Strains tested were wild-type (ATCC42720), *cnb1* mutants (YC198 and YC202) and *crz1* mutants (YC187 and YC467). (**C**) Doubling time of wild-type and calcineurin pathway mutants in 100% serum. * *P*<0.01, ** *P*<0.0001.

The survival and proliferation of fungal pathogens in host serum are essential to establish a successful bloodstream infection. In *C. albicans*, calcineurin but not Crz1 is essential for serum survival [26], [35]. In *C. lusitaniae*, we found that calcineurin is required for optimal growth on solid serum agar (50% serum, 2% agar) and liquid 100% serum (Figures 3A and 3B). Interestingly, we found that the optimal growth in liquid 100% serum is mediated by calcineurin-dependent Crz1 signaling in *C. lusitaniae* because *crz1* mutants exhibit intermediate growth kinetics between wild-type and calcineurin mutants (Figures 3B and 3C). This phenotype is distinct from *C. albicans* since *C. albican*s *crz1/crz1* mutants exhibited wild-type growth in serum. The doubling time of the *C. lusitaniae* wild-type strain in 100% serum at 37°C was 2.5 h, while the calcineurin mutants have a 6 h doubling time (P<0.0001; Figure 3C). The *crz1* mutants have an intermediate 3.1 h doubling time, which is significantly different from both the wild-type and the *cnb1* mutants (P<0.01; Figure 3C).

### Crz1 Plays a Greater Role than Calcineurin in Controlling Ca^2+^ Homeostasis

Calcium is one of the most important and abundant cations in living cells for the conduction of nerve impulses, normal clotting of blood (mammalian), and for signal transduction and growth (microorganisms). In several fungal pathogens, including *C. albicans* [36], [37], *C. neoformans* [38], *A. fumigatus* [20], and *Magnaporthe oryzae* [39], calcineurin is required for Ca^2+^ ion homeostasis. In *C. albicans*, *crz1/crz1* mutants exhibited intermediate Ca^2+^ sensitivity between wild-type and *cna1/cna1* mutants [18], [35], [40], suggesting calcineurin controls Ca^2+^ tolerance via Crz1. It has been shown that calcineurin is required for *C. albicans* survival during Ca^2+^ stress in serum, and is thus important for virulence in a murine systemic infection model [41]. In contrast, Brand et al. showed that Ca^2+^-driven thigmotropism is mediated by Crz1, but not calcineurin, in *C. albicans*, indicating that Crz1 might play a greater role in Ca^2+^ homeostasis in terms of contact responses [42].

**Figure 4:**
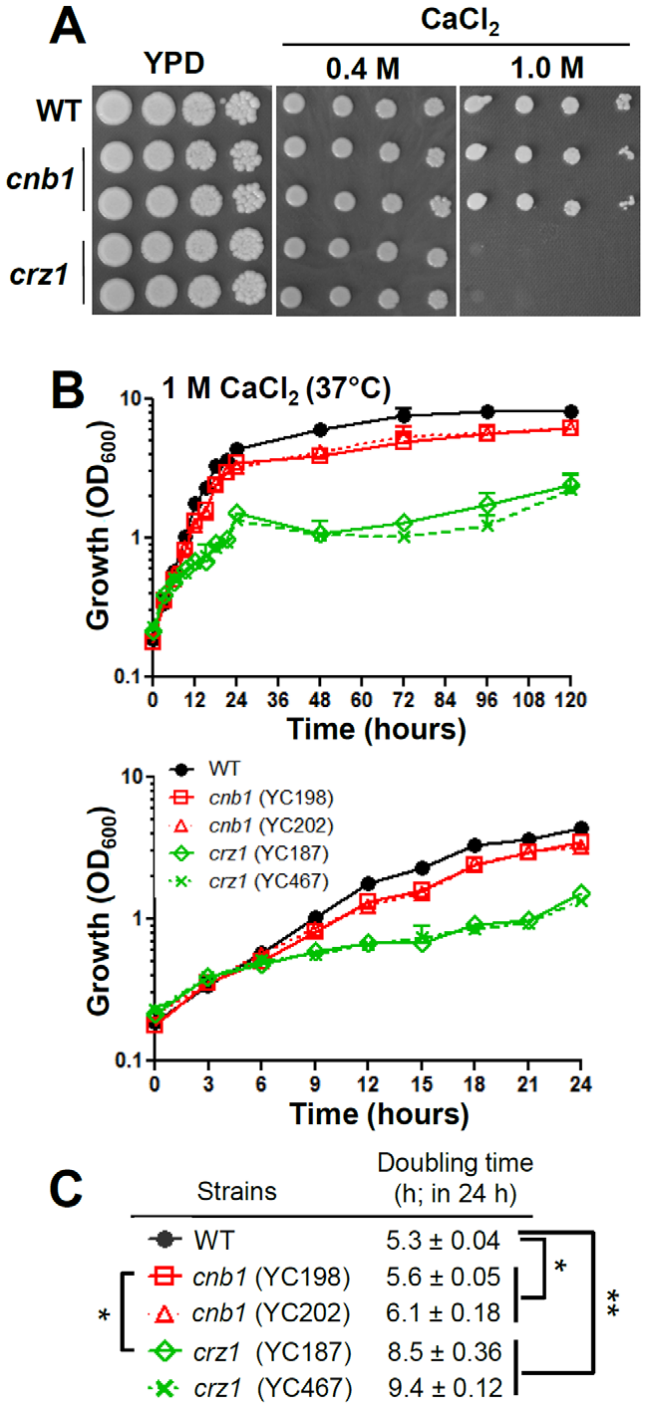
Transcription factor Crz1 plays a greater role than calcineurin in controlling Ca^2+^ ion homeostasis in *C. lusitaniae*. (**A**) Cells were grown overnight in YPD at 30°C, 5-fold serially diluted, and spotted onto YPD medium with or without CaCl_2_ at the concentrations indicated, and incubated at 37°C for 48 h. (**B**) The growth kinetics of *C. lusitaniae* wild-type and mutant strains on YPD containing 1 M CaCl_2_ at 37°C. Cells were grown overnight at 30°C, washed twice with dH_2_O, diluted to 0.2 OD_600_/ml in fresh liquid YPD medium, and incubated at 37°C with shaking at 250 rpm. The OD_600_ of cultures was measured at 0, 3, 6, 9, 12, 15, 18, 21, 24, 48, 72, 96, and 120 h (upper panel). The lower panel shows the growth kinetics between 0 and 24 h extracted from the upper panel. The experiments were performed in triplicate, and data was plotted using Prism 5.03. Strains tested were wild-type (ATCC42720), *cnb1* mutants (YC198 and YC202), and *crz1* mutants (YC187 and YC467). (**C**) Doubling time of wild-type and calcineurin pathway mutants in 1 M CaCl_2_. *P = 0.0002, **P<0.0001.

Here, we demonstrate that *C. lusitaniae* Crz1 plays important roles in controlling Ca^2+^ homeostasis because *crz1*, but not calcineurin, mutants exhibit Ca^2+^ sensitivity on solid YPD medium containing 1 M CaCl_2_ (Figure 4A), suggesting that Crz1 positively regulates Ca^2+^ tolerance via calcineurin-independent signaling (Figure 8). In liquid 1 M Ca^2+^-containing media, *crz1* mutants proliferate slowly with a doubling time of ∼9 h (P<0.0001) compared with the wild-type doubling time of 5.3 h (Figures 4B and 4C). Interestingly, we found that the *cnb1* mutants exhibit an intermediate growth rate (doubling time ∼5.9 h) between wild-type and *crz1* mutants in liquid medium containing 1 M CaCl_2_ (Figures 4B and 4C). These results are distinct from that found in *C. albicans*, suggesting divergence in the Ca^2+^ homeostasis roles of calcineurin and Crz1 in *C. albicans* and *C. lusitaniae*.

In *C. glabrata*, *crz1* mutants also exhibit sensitive growth in the presence of Ca^2+^ compared with the wild type, while calcineurin mutants show modestly resistant growth [16]. Thus, the roles of Crz1 in Ca^2+^ homeostasis seem to be conserved between *C. glabrata* and *C. lusitaniae*. However, calcineurin roles in Ca^2+^ response have diverged between the two *Candida* species.

### Calcineurin Controls Virulence in a Murine Systemic Infection Model

**Figure 5:**
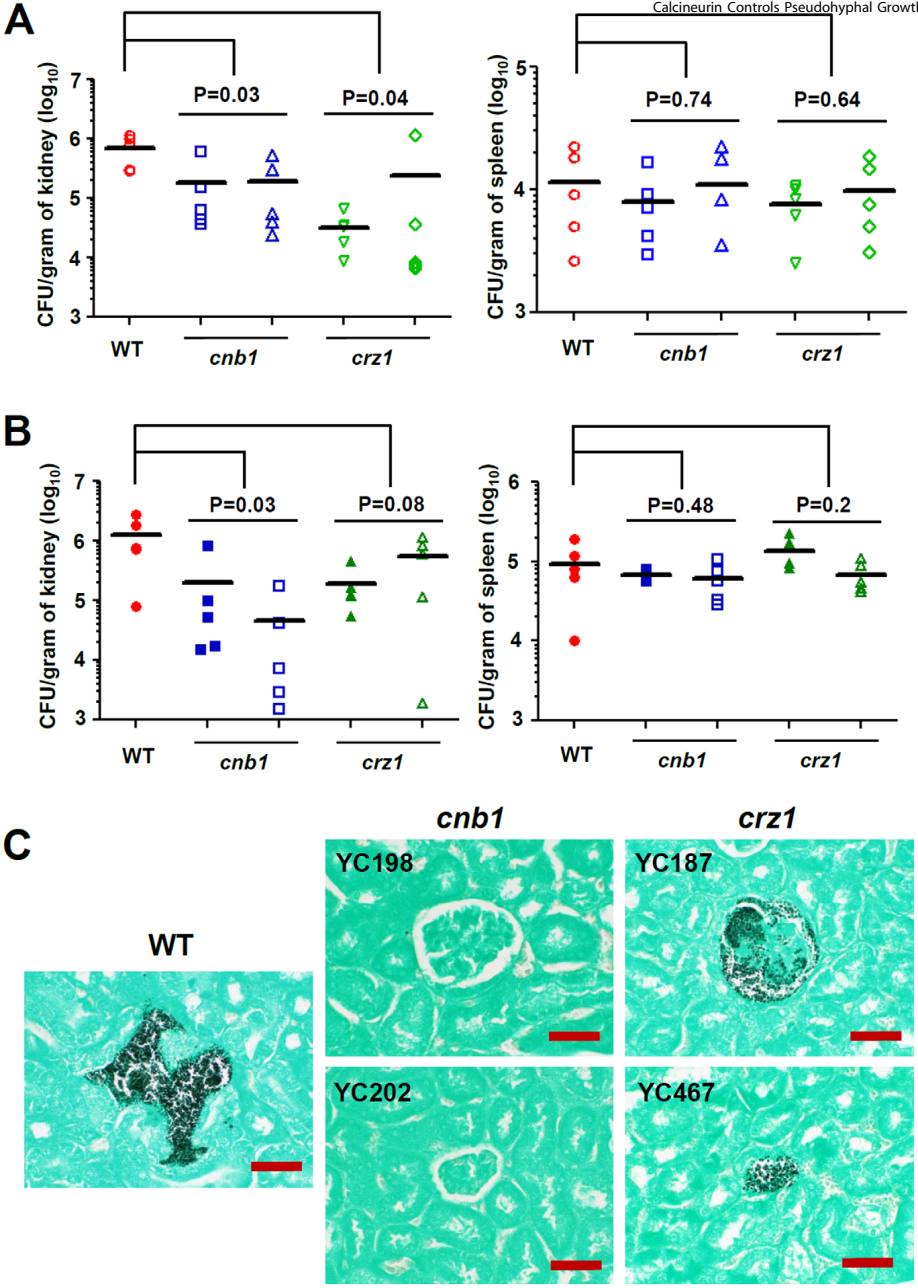
Calcineurin contributes to kidney tissue colonization in a murine systemic infection model. (**A**) The fungal burden in the kidneys and spleen of immunocompetent mice was determined at day 14 after challenge with 10^7^ yeast cells via lateral tail vein injection. Strains tested were wild-type (ATCC42720), *cnb1* mutants (YC198 and YC202), and *crz1* mutants (YC187 and YC467). The *P* value (ANOVA, Dunnett’s Multiple Comparison) between wild-type and mutants is shown. (**B**) The fungal burden in the kidneys and spleen of immunocompromised mice (cyclophosphamide-treated) was determined at day 7 after challenge with 10^7^ yeast cells via lateral tail vein injection. The *P* value (ANOVA, Dunnett’s Multiple Comparison) between wild-type and mutants is shown. (**C**) Histopathological sections of kidneys dissected from immunocompromised mice infected with wild-type, *cnb1*, or *crz1* mutant strains. The mice were challenged with 10^7^ cells and sacrificed at day 7. Gomori Methenamine Silver stain was used to observe *C. lusitaniae* colonization (black dots). Bar = 50 µm.

The roles of specific genes in virulence of *C. lusitaniae* have not been determined previously. *C. lusitaniae* is a less pathogenic *Candida* species compared to *C. albicans*. In contrast to *C. glabrata, C. lusitaniae* preferentially colonizes the kidney rather than splenic tissues in both immunocompetent (Figure 5A) and immunocompromised (Figure 5B) murine systemic infection models. In kidney tissues of immunocompetent mice at 14 days post-infection, calcineurin and *crz1* mutants exhibited a 3.8-fold (P = 0.03; ANOVA, Dunnett’s Multiple Comparison Test) and a 5.2-fold (P = 0.04) reduced fungal burden compared with the wild-type strain (Figure 5A). However, there was no difference between wild-type and calcineurin or *crz1* mutants in colonization of the spleen (Figure 5A), suggesting calcineurin signaling contributes to renal, but not splenic tissue colonization. This suggests that immune cells in the spleen might attack wild-type and calcineurin pathway mutants with similar mechanisms.

In kidney tissues of immunocompromised mice (cyclophosphamide-induced) at 7 days post-infection, calcineurin and *crz1* mutants exhibited 10-fold (P = 0.03) and 3.4-fold (P = 0.08) reduced fungal burdens compared with the wild-type strain (Figure 5B). However, there was no difference in splenic tissue fungal burden between the wild-type and mutants (right panel, Figure 5B).

In histopathological analysis of the immunocompromised murine model at day 7 post-infection, GMS-stained kidney tissues revealed no observable colonization by the calcineurin mutants (Figure 5C). The wild-type and *crz1* mutants proliferate as the yeast form, and no pseudohyphae or hyphae were found in the kidney (Figure 5C). In H&E staining, *C. lusitaniae* wild-type, calcineurin, and *crz1* mutants did not cause obvious tissue damage or necrosis (data not shown), suggesting that *C. lusitaniae* is a less pathogenic *Candida* species.

In *C. dubliniensis*, reduced hyphal formation is associated with attenuated virulence compared with *C. albicans* [43]. Although *C. lusitaniae* is able to form pseudohyphae *in vitro*, our demonstration that *C. lusitaniae* cannot form pseudohyphae *in vivo* is in accordance with *C. lusitaniae* being a less pathogenic yeast compared with *C. albicans* or *C. dubliniensis*. Our results indicate that hyphal growth might be an evolved virulence factor for *Candida* species that are common commensals.

### *C. lusitaniae* ATCC42720 is Less Pathogenic in a Murine Keratitis Model than *C. albicans* SC5314

To date, reports indicating *C. lusitaniae* as the etiologic agent of fungal keratitis in humans are limited. Two clinical keratitis reports available that involve *C. lusitaniae* also included other *Candida* species or fungal pathogens in a polymicrobial keratitis in which a clear causal participation of *C. lusitaniae* at the onset or in the infectious course could not be clearly established [44], [45].

In this report, we determined the role of calcineurin signaling in corneal virulence of *C. lusitaniae* by comparing the disease severity induced by calcineurin and *crz1* mutants versus the wild-type strain (ATCC42720). We initially tested the wild-type strain of *C. lusitaniae* in immunocompetent ICR mice using a previously described murine experimental keratitis procedure [15]; however, none of the test mice including those inoculated with the wild-type strain developed basic clinical features of keratitis such as corneal opacity, inflammation, surface irregularities, or ulcerations. To increase the predisposition of test mice to ocular infection from this rare opportunistic yeast pathogen, we administered cyclophosphamide before inoculating traumatized corneas with two doses of fungal inoculum (10^6^ CFU/dose).

In contrast to 100% keratitis (10/10 mice) caused by *C. albicans* wild-type, the *C. lusitaniae* wild-type induced keratitis in only 3/18 (16.7%) mice inoculated on consecutive days with 10^6^ yeast cells (Figure S5A). The disease score of the pooled mice showing clinical indications of keratitis was mild (1^st^ day, mean score = 4.00±2.00), and only persisted until the 3^rd^ day post inoculation (0.67±1.15, Figure S5B). Mice had already recovered from the disease on the 4^th^ day post inoculation and regained the naive appearance of their eyes. Notably, none of the independently derived *cnb1* and *crz1* mutant strains were able to induce persistent keratitis (Figure S5). Histological staining of the cornea infected with wild-type or mutants did not reveal obvious yeast or pseudohyphae (data not shown).

In *C. albicans* experimental keratitis, mutants that are deficient in genes which regulate morphogenesis from yeast to filamentous form have virulence that is fully or partially attenuated, demonstrating an important role played by filamentation in the pathogenesis of *C. albicans* keratitis [46]. Thus, the low virulence of *C. lusitaniae* in post-traumatized corneas of immunosuppressed mice may be attributable to its failure to form pseudohyphae during infection.

### Calcineurin Controls Drug Tolerance in *C. lusitaniae*

**Figure 6:**
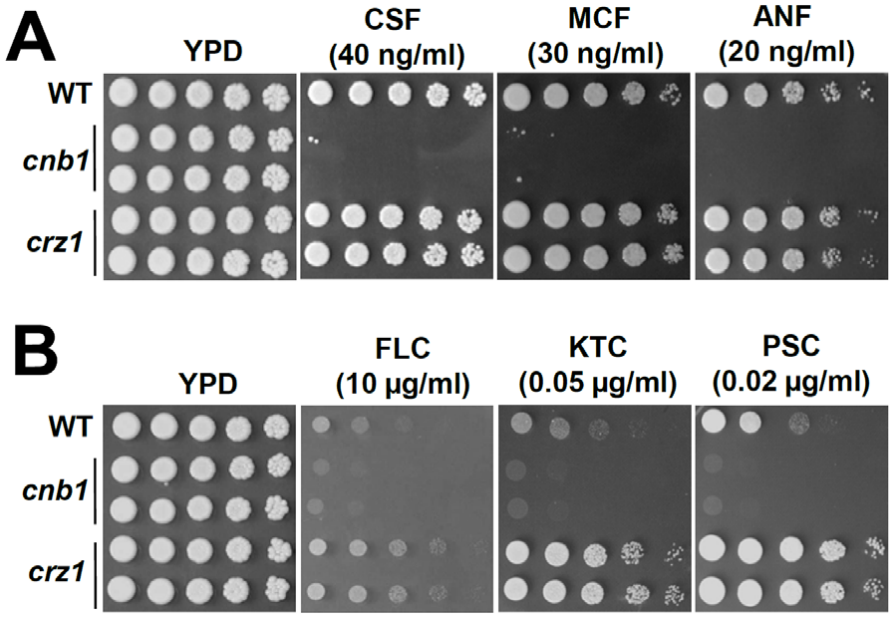
Calcineurin controls antifungal drug tolerance in *C. lusitaniae*. Cells were grown overnight in YPD at 30°C, 5-fold serially diluted, and spotted onto YPD medium containing echinocandins (**A**) or azoles (**B**) at the indicated concentrations, and incubated at 30°C for 48 h.

*C. albicans* calcineurin and *crz1* mutants are known to be susceptible to azole antifungal agents. Furthermore, calcineurin inhibitors and fluconazole have been shown to exhibit synergistic antifungal activity against *C. albicans*. We used spot assays and E-tests [47], [48] to compare drug tolerance between wild-type and calcineurin or *crz1* mutant strains. With spotting assays, *C. lusitaniae cnb1* mutants exhibited severe growth defects compared to the wild-type in the presence of echinocandins (caspofungin, micafungin, and anidulafungin) (Figure 6A and Figure 8) and to a somewhat lesser extent with azoles (fluconazole, ketoconazole, and posaconazole) (Figure 6B). This is consistent with results of the E-test as *cnb1* mutants exhibit lower MICs for caspofungin (12 fold) and the azoles (2 fold) compared with the wild-type (Table 1). For the roles of calcineurin and Crz1 in amphotericin B tolerance, we found that there is no difference between the wild-type and calcineurin or *crz1* mutants via E-tests (Table 1).

**Table 1:** Calcineurin is required for drug tolerance in *C. lusitaniae*.

Strain	MIC or Range (µµg/ml)#				
	Caspofungin	Ketoconazole	Voriconazole	Fluconazole	Amphotericin B
ATCC42720 (WT)	0.38	0.032	0.016	1.0	0.19
*cnb1* (YC198)	**0.032**	**0.016**	**0.008**	0.5	0.19
*cnb1* (YC202)	**0.032**	**0.016**	**0.008**	0.5	0.19
*crz1* (YC187)	0.38	0.064	0.023–0.032	1.0	0.125
*crz1* (YC467)	0.38	0.064	0.023–0.032	1.0–1.5	0.125

# Cells were grown overnight at 30°C and washed twice with dH_2_O. Then 0.5 OD (in 500 µl) of cells was spread on RPMI 1640 media (Remel; R04067). After 20 min, the E-test strips (bioMérieux Corp.) were transferred to the surface of the media. The minimum inhibitory concentrations (MIC) were read after 24 h incubation at 35°C according to the manufacturer’s instructions.

Interestingly, *C. lusitaniae crz1* mutants exhibited increased tolerance to azoles (Figure 6B), which is different from *C. albicans* and *C. dubliniensis* [15], but similar to *C. glabrata crz1* mutants [16]. The results suggest that *C. lusitaniae* Crz1 negatively regulates azole tolerance and it is independent of calcineurin (Figure 8). Unlike intermediate echinocandin tolerance phenotypes of *crz1/crz1* mutants in *C. albicans* and *C. dubliniensis* and *crz1* mutants in *C. glabrata*, *C. lusitaniae crz1* mutants exhibit no difference in comparison to wild-type (Figure 6A). These findings were supported by the data from the roles of Crz1 on cell wall integrity (Figure 1A).

### Calcineurin Inhibitor Shows Synergistic Antifungal Activity with Caspofungin Against Clinical Echinocandin-resistant *C. lusitaniae* Strains

**Figure 7:**
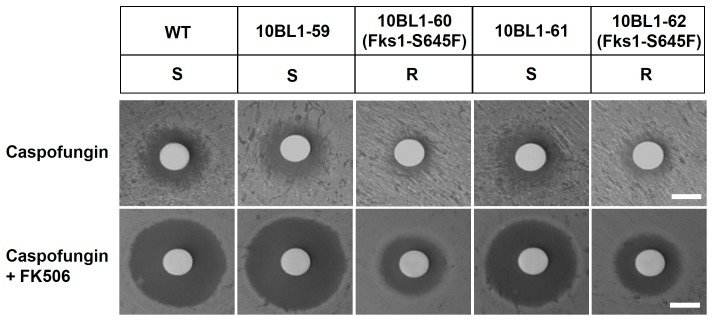
Calcineurin inhibitor exhibits synergistic antifungal activity with caspofungin against *C. lusitaniae* wild-type and echinocandin-resistant strains. Disk diffusion assays were used to determine synergistic antifungal activity with caspofungin against clinical echinocandin-resistant *C. lusitaniae* strains. Cells were grown overnight at 30°C, and 0.1 OD_600_ (in 100 µl) was spread on the surface of RPMI media lacking or containing FK506 (1 µg/ml). A disk was placed on the surface of the medium and 12.5 µg caspofungin (5 µl of 2.5 mg/ml) was added to each disk. The plates were incubated at 30°C for 48 h and photographed. S = caspofungin-sensitive; R = caspofungin-resistant (less susceptible). Scale bar = 6 mm.

Recently, Desnos-Ollivier reported two clinical echinocandin resistant *C. lusitaniae* strains, which harbor a point mutation (S645F) at the Fks1 hot spot region 1 [8]. Based on our findings that calcineurin is required for echinocandin tolerance, we tested whether the calcineurin inhibitor FK506 reverses echinocandin resistance via disk diffusion and checkerboard assays. As seen in Figure 7 (upper panel), two echinocandin resistant isolates (10BL1-60 and 10BL1-62) are resistant to caspofungin (as evidenced by smaller halos) compared with the wild-type and two clinical echinocandin sensitive isolates (10BL1-59 and 10BL1-61). The addition of the calcineurin inhibitor FK506 enhanced the antifungal activity of caspofungin (as seen by an increased halo diameter compared with no FK506 treatment) against both echinocandin resistant isolates (Figure 7, lower panel). The results found in disk diffusion assays are supported by checkerboard assays in which FK506 exhibited synergistic antifungal activity with caspofungin against echinocandin-sensitive and -resistant isolates (FIC <0.5; Table 2).

**Table 2:** Calcineurin inhibitor FK506 exhibits synergistic antifungal activity with caspofungin against echinocandin resistant strains.

Strain#	MIC or Range (µµg/ml)					
	MIC_50_ alone		MIC_100_ alone		MIC_100_ combined	FIC&
	CSF	FK506	CSF	FK506	CSF/FK506	CSF/FK506
ATCC42720 (WT)	0.5	>4	1.0	>4	0.25/0.5	0.313
10BL1-59	0.5	>4	2.0	>4	0.25/0.25	0.156
10BL1-60*	4.0	>4	8.0	>4	2.0/1.0	0.375
10BL1-61	1.0	>4	2.0	>4	0.5/0.063	0.258
10BL1-62*	4.0	>4	8.0	>4	2.0/0.25	0.281

# Strains were grown overnight with shaking at 30°C and washed twice in dH_2_O. The OD_600_ was taken of the cultures with a spectrophotometer and diluted to 0.01 OD_600_/ml in RPMI-1640 medium [Sigma R1383 (8.4g) and MOPS (34.5g) in 1L dH_2_O buffered to pH7 with NaOH]. Minimum inhibitory concentrations (MIC) of each drug alone and fractional inhibitory concentrations of the drugs in combination were determined using the broth microdilution method according to the Clinical and Laboratory Standards Institute (CLSI) protocol M27-A3. Final concentrations of caspofungin (CAS) ranged from 16 to 0.0312 µg/ml. FK506 concentrations ranged from 4.0 to 0.063 µg/ml.

& FIC index = (MIC_combined_ drug 1/MIC _alone_ drug 1) + (MIC_combined_ drug 2/MIC_alone_ drug 2) FIC ≤0.5 (synergy), >0.5 but <1.0 (additive), >1.0 but ≤2.0 (no interaction), >2.0 (antagonism).

* *C. lusitaniae* clinical echinocandin resistant isolates with the Ser645Phe mutation in the Fks1 protein.

**Figure 8 pone-0044192-g008:**
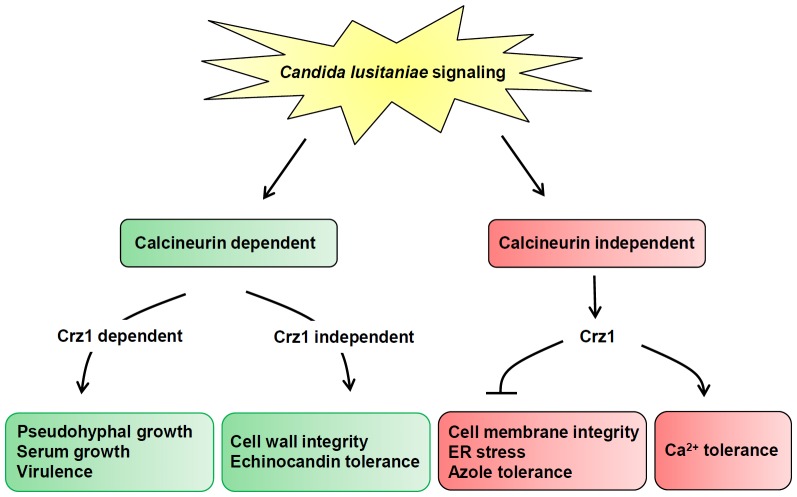
Proposed roles of calcineurin and Crz1 in core stress responses in *C. lusitaniae*. *C. lusitaniae* core stress responses including pseudohyphal growth, drug tolerance, virulence, serum growth, cell membrane and wall integrity, ER stress, and Ca^2+^ homeostasis are controlled by either calcineurin-dependent or -independent signaling cascades. The pseudohyphal development, serum growth, and virulence are controlled by Crz1-mediated calcineurin signaling, while cell wall integrity and echinocandin tolerance are governed by Crz1-independent calcineurin signaling (green shading). Crz1 also exhibits calcineurin-independent functions to: 1) negatively regulate cell membrane integrity, ER stress, and azole tolerance; 2) positively regulate Ca^2+^ tolerance (red shading).

We summarize the roles of calcineurin and Crz1 in core stress responses in *C. lusitaniae* signaling (Figure 8)[Fig pone-0044192-g008]. In the future, it will be important to investigate if calcineurin inhibitors exhibit *in vivo* synergistic antifungal activity against *C. lusitaniae* wild-type and drug-resistant isolates. However, due to immunosuppression as a result of calcineurin inhibitor action on mammalian calcineurin, development of reduced or non-immunosuppressive calcineurin inhibitors may be required. Before this, tests of caspofungin’s therapeutic effects on calcineurin mutant infected mice could be performed in the future to address if a calcineurin inhibitor might be synergistic *in vivo* with caspofungin against *C. lusitaniae* wild-type and drug-resistant strains.

## Materials and Methods

### Ethics Statement

Animals studies conducted in the Division of Laboratory Animal Resources (DLAR) facilities at Duke University Medical Center (DUMC) were handled as defined by the United States Animal Welfare Act and in full compliance with the guidelines of the DUMC Institutional Animal Care and Use Committee (IACUC). The murine systemic infection model was reviewed and approved by the DUMC IACUC under protocol number A238-09-08.

Murine ocular infection studies conducted at the animal house facility of the Department of Microbiology and Parasitology, University of Perpetual Help-Dr. Jose G. Tamayo Medical University (UPH-DJGTMU), were performed in accordance with the ARVO Statement for the Use of Animals in Ophthalmic and Vision Research, the United States Animal Welfare Act, and the Republic of the Philippines Animal Welfare Act of 1998 (RA No. 8485), and in full conformity with the guidelines set forth in the UPH-DJGTMU research manual. The protocol was formally approved by the UPH-DJGTMU institutional review board after review by the Regional Institute for Tropical Medicine Institutional Animal Care and Use Committee (RITM - IACUC) under research protocol number No. 010. All animal infection experiments were conducted by properly trained personnel, including licensed veterinarians.

### Yeast Strains, Media, and Chemicals

Fungal strains used in this study are listed in Table 3. The following media were used in this study: yeast extract peptone dextrose (YPD; 1% yeast extract, 2% peptone, 2% glucose) liquid medium and agar (2%) plates; serum agar (10% or 50% serum, 2% agar); filament agar (FA; 1.7 g yeast nitrogen base without amino acids and without ammonium sulfate, 5 g glucose, 40 g bacto agar in 1 L dH_2_O); and potato dextrose agar (PDA; 1∶10 dilution PDA, 14.5 g of agar in 1 L dH_2_O). YPD medium containing 100 µg/ml nourseothricin was used to select transformants. The following supplements were added to the media at the concentrations indicated: FK506 (Astellas Pharma Inc.), cyclosporin A (CsA, LC Laboratories), sodium dodecyl sulfate (Fisher), calcofluor white (fluorescent brightener 28, Sigma), congo red (Sigma), tunicamycin (Sigma), dithiothreitol (Sigma), fetal bovine serum (Invitrogen), calcium chloride (Sigma), fluconazole (Bedford Laboratories), posaconazole (Merck), ketoconazole (Sigma), caspofungin (Merck), micafungin (Astellas Pharma Inc.), and anidulafungin (Pfizer Inc.).

**Table 3:** Strains used in this study.

Strain	Genotype	Background	Reference
*Candida lusitaniae*			
ATCC42720	Prototrophic wild-type; genome sequence reference strain	Clinical isolate	[6], [28]
YC198^a^	*cnb1*Δ*::SAT1*	ATCC42720	This study
YC202^a^	*cnb1*Δ*::SAT1*	ATCC42720	This study
YC187^b^	*crz1*Δ*::SAT1*	ATCC42720	This study
YC467^b^	*crz1*Δ*::SAT1*	ATCC42720	This study
10BL1-59	Fks1 hot spot 1 wild-type	Clinical isolate	[8]
10BL1-60	Fks1 hot spot 1 S645F	Clinical isolate	[8]
10BL1-61	Fks1 hot spot 1 wild-type	Clinical isolate	[8]
10BL1-62	Fks1 hot spot 1 S645F	Clinical isolate	[8]
*Candida albicans*			
SC5314	Prototrophic wild-type	Clinical isolate	[50]

a two independent *cnb1* mutants.

b two independent *crz1* mutants.

### Strain Construction

The *C. lusitaniae CNB1* and *CRZ1* genes were disrupted with the disruption cassette carrying the nourseothricin resistance gene *SAT1* [49]. For the *CNB1* gene disruption, approximately 0.85 kb 5′ (amplified with primers JC21/JC22; Table 4) and 3′ (amplified with primers JC23/24) noncoding regions flanking the *CNB1* open reading frame were PCR amplified from genomic DNA of the wild-type strain ATCC42720. The 1.8 kb *SAT1* gene was amplified from plasmid pSFS2A with primers JC8/JC9. All three PCR products were then treated with ExoSAP-IT (USB Corp. Cat#78201) to remove contaminating primers and deoxynucleotide triphosphates (dNTPs). The resultant ExoSAP-IT treated products were combined in a 1∶3:1 molar ratio (5′*CNB1* ^NCR^-*SAT1*-3′*CNB1* ^NCR^) to generate the disruption cassette by overlap PCR using flanking primers JC25/JC26 (∼100 bp closer to the *CNB1* ORF compared to JC21/JC24, respectively). Overlap PCR resulted in a ∼3.5 kb 5′*CNB1* ^NCR^-*SAT1*-3′*CNB1* ^NCR^ disruption allele. The *CNB1* gene was disrupted in the wild-type ATCC42720 strain by transformation with 0.2 to 1 µg of gel-purified disruption DNA using the Yeast EZ Transformation Kit II (Zymo Research, Orange, CA) and plated on YPD medium containing 100 µg/ml nourseothricin to select for mutants that have integrated the *SAT1* cassette. Disruption of the *CRZ1* gene used a similar approach whereby approximately 1 kb 5′ (amplified with primers JC31/JC32) and 3′ (amplified with primers JC33/34) noncoding regions of the *CRZ1* open reading frame were PCR amplified from genomic DNA of the wild-type ATCC42720 strain. The two PCR products were treated with ExoSAP-IT and combined in a 1∶3:1 molar ratio (5′*CRZ1* ^NCR^-*SAT1*-3′*CRZ1* ^NCR^) with the amplified *SAT1* to generate the disruption PCR cassette by overlap PCR using primers JC35/JC36. The *CRZ1* gene was disrupted in the wild-type ATCC42720 strain using similar yeast transformation methods. Two independent nourseothricin-resistant *cnb1* mutants as well as two independent *crz1* mutants derived from separate transformations were obtained. Mutants were confirmed by PCR and Southern blot analysis.

**Table 4:** PCR primers used in this study.

Primer	Use	Sequence (5′ 3′)
JC8	*SAT1* marker	CGTCAAAACTAGAGAATAA
JC9	*SAT1* marker	AGGACCACCTTTGATTGT
JC21	5′NCR of *CNB1*	AAGCAAAAGGCGTGTGATGA
JC22	5′NCR of *CNB1*	TCGTTTTCTTTATTATTCTCTAGTTTTGACG TGTTGTCAGGTGTATCGTTGG
JC23	3′NCR of *CNB1*	GTGGTAATTATTACTATTTACAATCAAAGGTGGTCCT GCTGTGGAACTCCTATGGATT
		
JC24	3′NCR of *CNB1*	GAAAGAAAATCGTGCGGATC
JC25	*CNB1* overlap	TGCGAGAAGTTCTTCACTCAA
JC26	*CNB1* overlap	AAATATCCAGCCAGGTGGAA
JC27	*CNB1* ORF	GGTTGGCGTCATCTAAAGTGT
JC28	*CNB1* ORF	CGTCAAAACATTGGCTACCA
JC31	5′NCR of *CRZ1*	TGATAATAGCATAGCCTCGCA
JC32	5′NCR of *CRZ1*	TCGTTTTCTTTATTATTCTCTAGTTTTGACG GAGAAAGGCGCACTAGAAGAA
JC33	3′NCR of *CRZ1*	GTGGTAATTATTACTATTTACAATCAAAGGTGGTCCT ACAAGAAATATCTGGAGGTTC
		
JC34	3′NCR of *CRZ1*	CACTAGCTGAATCTAGCGAA
JC35	*CRZ1* overlap	CTATGGTACTTTTTTCTTTGG
JC36	*CRZ1* overlap	AATGTGACGAACTGCGACAA
JC37	*CRZ1* ORF	CGAATATGGCCCCTCTGAAC
JC38	*CRZ1* ORF	TTGGCCCATTTGGTGAAGAT

Sequences complementary to the *SAT1* marker are underlined.

### Spot Growth Assays

Cells were grown overnight at 30°C, washed twice with dH_2_O, and the OD at 600 nm was measured. Cells were resuspended into an appropriate amount of dH_2_O to achieve 1 OD_600_/ml. Three microliters of five-fold serial dilutions (40 µl of 1 OD_600_/ml cells plus 160 µl of dH_2_O as the first dilution in a 96-well plate) from each strain were spotted with a multichannel pipette onto solid media. The plates were then incubated at the indicated temperatures for 48 hr and photographed.

### Doubling Time Measurement

The doubling time was calculated by using formula T * ln2/(ln(OD^T^/OD^T0^) where OD^T^ and OD^T0^ represent OD_600_ at time T and initial time (T_0_), respectively. The log phase time points were chosen from 0 to 9 h (100% serum) or 0 to 30 h (in 1 M CaCl_2_).

### Disk Diffusion Assays

Cells were grown overnight at 30°C, and 0.1 OD_600_ (in 100 µl) was spread on the surface of YPD medium in the absence or presence of FK506 (1 µg/ml). A blank paper disc (6 mm; BD Cat#231039) was placed on the surface of the medium, and 5 µl of caspofungin (12.5 µg) or 5 µl of DMSO (as control) were added to each disk. The plates were incubated at 30°C for 48 h and photographed.

### Scanning Electron Microscopy

The culture colonies were excised from the agar and fixed in 3% glutaraldehyde in 0.1 M Na cacodylate buffer, pH 6.8 for several days at 4°C. They were then rinsed in three 30-minute changes of cold 0.1 M Na cacodylate buffer, pH 6.8 followed by a graded dehydration series of 2-hour changes in cold 30% and 50% EtOH and held overnight in 70% EtOH. Dehydration was completed with 1 hour changes of cold 95% and 100% EtOH at 4°C warming to room temperature in the 100% EtOH. Two additional 1 hour changes of room temperature 100% EtOH completed the dehydration series. The samples were then critical point dried in liquid CO_2_ (Samdri-795, Tousimis Research Corp., Rockville MD) for 15 minutes at critical point. The agar pieces were mounted on stubs with double stick tape, pressed down completely around the edge, and then sealed with silver paint to ensure good conductivity. Samples were sputter coated with 50Å of Au/Pd (Hummer 6.2, Anatech U.S.A., Hayward CA). Samples were held in the vacuum desiccator until viewed with a JEOL JSM 5900LV SEM at 15 kV.

### Calcofluor White Staining

*C. lusitaniae* wild-type strain (ATCC42720) was grown on V8 (pH = 7) solid plate at 37°C for 7 days. Filamentous cells on the edge of the colony were excised with a sterile scalpel and mixed with 100 µl of calcofluor white solution (1 mg/ml) and incubated for 5 minutes at 24°C. The cell mixtures were washed three times with 1 ml of dH_2_O, and then resuspended in 100 µl of dH_2_O. Five microliters of the stained cell suspension were spotted onto a slide coated with poly-L-lysine (Polysciences Cat#22247). The cells were visualized at 1,000X magnification under bright field and UV/DAPI, and photographed.

### Murine Systemic Infection Model

Five- to six-week-old male CD1 mice from Jackson Laboratories (n = 5 for each group) were used in this study. For the immunocompromised mouse model, each mouse received 4 doses of cyclophosphamide (Sigma Cat#C7397; 150 mg/kg in 200 µl) at day -4 and -1 before and day 2+ and 5+ after *C. lusitaniae* infection. *C. lusitaniae* strains were grown in 5 ml YPD overnight at 30°C with shaking at 250 rpm. Cultures were washed twice with 10 ml of phosphate buffered saline (PBS), and the cells were then resuspended in 2 ml of PBS. Cells were counted with a hemocytometer and resuspended in an appropriate amount of PBS to obtain an infection inocula of 5×10^7^ cells/ml. Appropriate dilutions of the cells were plated onto YPD and incubated at 30°C for 48 hr to assess cell viability. Two hundred microliters (10^7^ cells) were used to infect mice by lateral tail vein injection.

To determine fungal burden, mice were sacrificed at day 14 for immunocompetent mice and at day 7 for immunocompromised mice, and kidney and spleen tissues of *C. lusitaniae* infected animals were dissected. The organs were weighed, transferred to a 15 ml Falcon tube filled with 5 ml PBS, and homogenized for 5 seconds at 17,500 rpm/min (Power Gen 500, Fisher Scientific). Tissue homogenates were serially diluted and 100 µl was plated onto YPD medium. The plates were incubated at 30°C for 48 hr to determine CFU per gram of organs. The identity of organ-recovered colonies was confirmed by colony PCR. For histopathological analysis, kidneys were excised at day 7 from immunocompromised mice, fixed in 10% phosphate-buffered formalin (Fisher), and Gomori Methenamine Silver (GMS) and Hematoxylin and Eosin (H&E) stainings were performed by the Department of Pathology at Duke University. After slide preparation, each sample was examined thoroughly by microscopy for analysis of *Candida* colonization (GMS) and tissue necrosis (H&E). Images were captured using an Olympus Vanox microscope at PhotoPath, Duke University Medical Center.

### Murine Ocular Infection Model

*Candida lusitaniae* wild-type (ATCC42720) and calcineurin pathway mutant strains, *cnb1* (YC198 and YC202) and *crz1* (YC187 and YC467), were grown in 100 mL YPD broth in an orbital shaker (200 rpm) for 24 hrs at 25°C. An aliquot of the culture (10 mL) was pelleted by centrifugation at 3,000 rpm for 10 minutes and then washed three times with sterile PBS (pH = 7.4). Cells were diluted using sterile PBS to a concentration approximately equal to 10^6^ CFU/5 µL. The concentration was determined by using a spectrophotometer that measured optical density by reading at a wavelength of 600 nm and multiplying it by a conversion factor of 1 OD_600_, equivalent to 3×10^7^ cells/mL. Cell density was verified by plating cells on YPD for 48 hr at 25°C. The virulent wild-type strain of *C. albicans* (SC5314) was used as the reference for the *C. lusitaniae* ocular infection study as this strain has been used extensively in various experimental keratitis reports**.**

Six- to eight week old outbred ICR mice (20–28 g) used in the study were from the Research Institute for Tropical Medicine (RITM), Alabang, Philippines. Mice were handled in accordance with the ARVO Statement for the Use of Animals in Ophthalmic and Vision Research and the animal protocol used in this study was approved by the University of Perpetual Help Institutional Review Board. The experimental keratomycosis protocol described previously [15] was performed with minor modifications to increase predisposition of test animals to corneal infection by *C. lusitaniae*. Briefly, mice were immunosuppressed with cyclophosphamide treatment (160–180 mg/kg) at -5, -3, and -1 day prior to inoculation. The first dose of fungal inoculum (10^6^ CFU) was introduced one day after the last administration of cyclophosphamide. Before introducing the first inoculum dose, mice were subject to general anesthesia by intramuscular injection of Zoletil 50 (10–15 mg/kg body weight; Virac, Australia) followed by topical application to the eyes with proparacaine hydrochloride ophthalmic solution (Alcaine®, Alcon-Couvreur, Belgium). Excess solution was removed with a sterile cotton swab. The eyes were then superficially scarified using a 25-gauge hypodermic needle and then inoculated with 5 µL of *Candida* culture (10^6^ CFU) or sterile PBS. Inoculum was spread evenly by rubbing the eye for a few seconds with the eyelid. The next dose of inoculum (10^6^ CFU) was applied 24 hours later with no additional scarification procedures performed in order to minimize discomfort to the animals. Animals were anesthetized with a lower dose of Zoletil 50 (5–10 mg/kg body weight) before introducing the second dose of fungal inoculum. Clinical scoring of fungal keratitis was visually assessed for 8 days following the second inoculum application. The visual scoring system employed in this study was based on three parameters: area of opacity, density of opacity, and surface irregularity. A grade of 0 to 4 was assigned on each of these criteria to yield a maximum score of 12. Mice were housed in comfortable cages with a constant supply of clean food and water, and cages were cleaned and sanitized daily with commercial disinfectant to avoid potential infection arising from other pathogens. Three mice at days 4 and 8 post infection were sacrificed by cervical dislocation. Eyes were removed and fixed in neutral formalin solution (10% formaldehyde in PBS) before submitting them to the laboratory for histological examination.

### Broth Microdilution Minimum Inhibitory Concentration (MIC) Assay

One wild-type *C. lusitaniae* strain (ATCC42720), two echinocandin-resistant, and two echinocandin-susceptible strains were grown overnight with shaking at 30°C and washed twice in dH_2_O. The OD_600_ was taken of the cultures with a spectrophotometer and diluted to 0.01 OD_600_/ml in RPMI 1640 medium (8.4 g of Sigma Cat# R1383 and 34.5 g of MOPS in 1 L dH_2_O buffered to pH 7 with sodium hydroxide). Minimum inhibitory concentrations of each drug alone and fractional inhibitory concentrations (FIC) of the drugs in combination were determined using the broth microdilution method according to the Clinical and Laboratory Standards Institute (CLSI) protocol M27-A3. Final concentrations of caspofungin ranged from 16 to 0.0312 µg/ml and FK506 concentrations ranged from 4.0 to 0.063 µg/ml. A FIC ≤0.5 was considered to be synergistic, 0.5 ∼ 1.0 was considered additive, while 1.0 ∼ 2.0 was considered no interaction.

### Statistical Analysis

Statistical analysis was conducted using Prism 5.03 software (GraphPad, La Jolla, Calif., USA). The significance of differences in fungal burdens was determined using one-way ANOVA, Dunnett’s Multiple Comparison Tests. The differences of doubling time were determined using unpaired t test and one-way ANOVA, Dunnett’s Multiple Comparison Tests. A *P* value of <0.05 was considered significant.

## Supporting Information

Figure S1 **Amino acid identity and pairwise alignment of calcineurin regulatory subunit (Cnb1) from** ***C. albicans*** **,** ***C. lusitaniae*** **, and** ***S. cerevisiae*** **.**
(TIF)

Figure S2 **Amino acid identity and pairwise alignment of calcineurin downstream target Crz1 from** ***C. albicans*** **,** ***C. lusitaniae*** **, and** ***S. cerevisiae*** **.**
(TIF)

Figure S3 **Few** ***C. lusitaniae*** **isolates exhibit temperature-sensitive growth when exposed to calcineurin inhibitors.**
(TIF)

Figure S4 ***C. lusitaniae*** **produced pseudohyphae growth on filament-inducing solid agar medium.**
(TIF)

Figure S5 **Clinical outcomes of ocular inoculations with** ***C. lusitaniae*** **wild-type (ATCC 42720),** ***cnb1*** **mutants (YC198 and YC202),** ***crz1*** **mutants (YC187 and YC467), or** ***C. albicans*** **wild-type (SC5314).**
(TIF)
