# Supplementary figures and images for: Calcineurin Is Required for Pseudohyphal Growth, Virulence, and Drug Resistance in Candida lusitaniae
Source: PLoS One. 2012 Aug 31;7(8):e44192. doi: 10.1371/journal.pone.0044192 (PMC3432075; doi:10.1371/journal.pone.0044192)

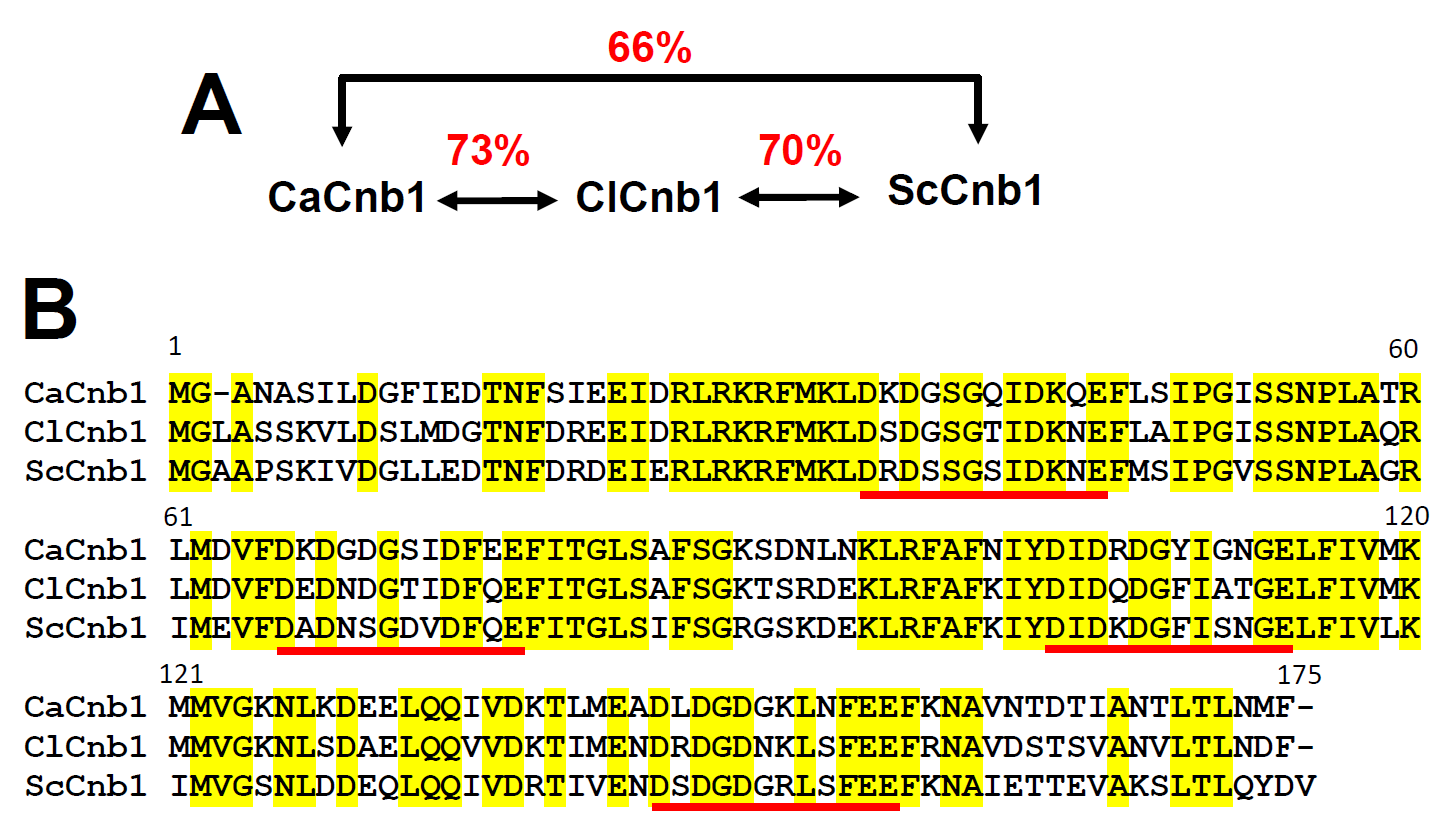

Supplement: Figure S1 — Amino acid identity and pairwise alignment of calcineurin regulatory subunit (Cnb1) from C. albicans , C. lusitaniae , and S. cerevisiae . (TIF) [file pone.0044192.s001.tif]

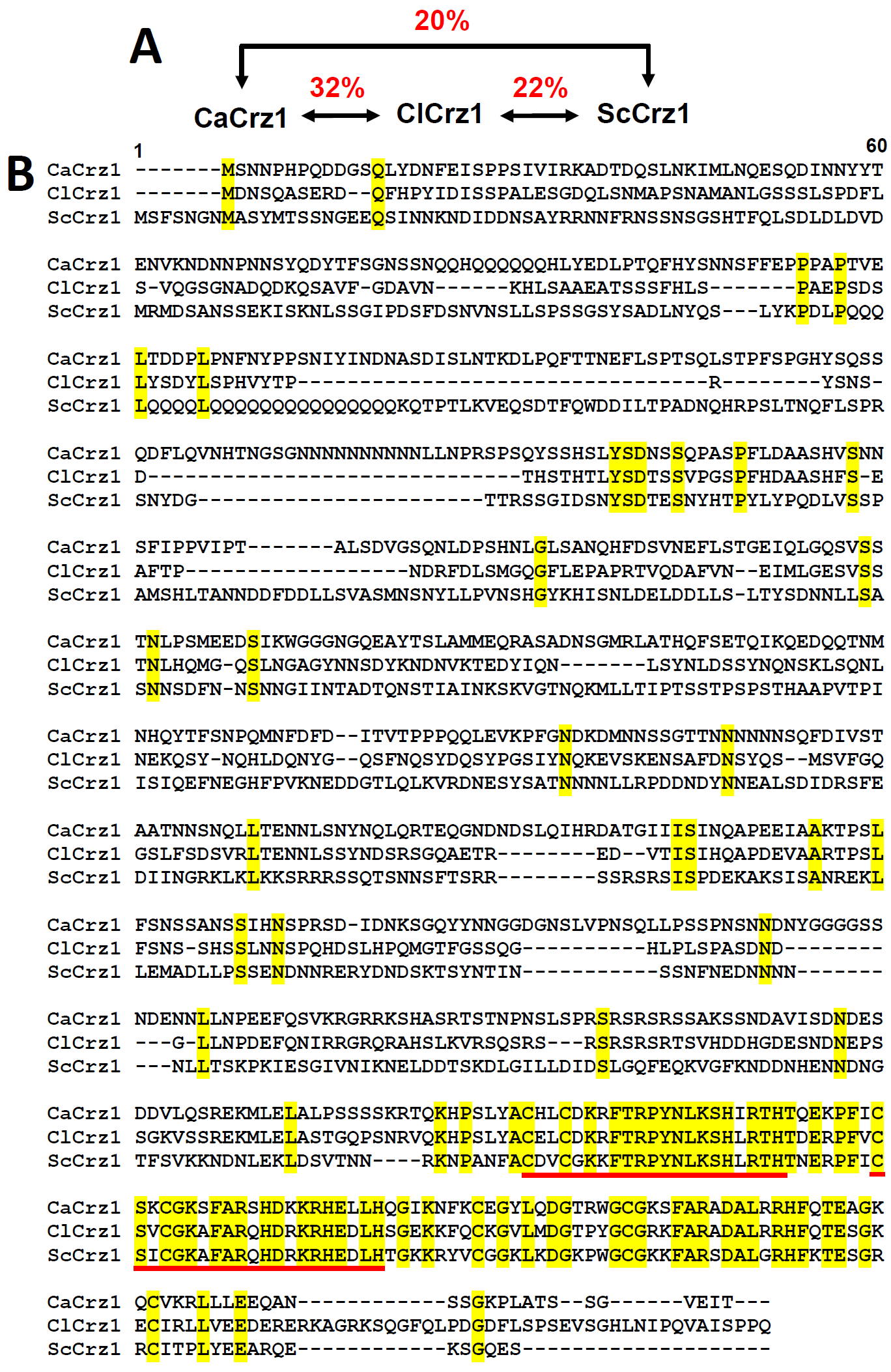

Supplement: Figure S2 — Amino acid identity and pairwise alignment of calcineurin downstream target Crz1 from C. albicans , C. lusitaniae , and S. cerevisiae . (TIF) [file pone.0044192.s002.tif]

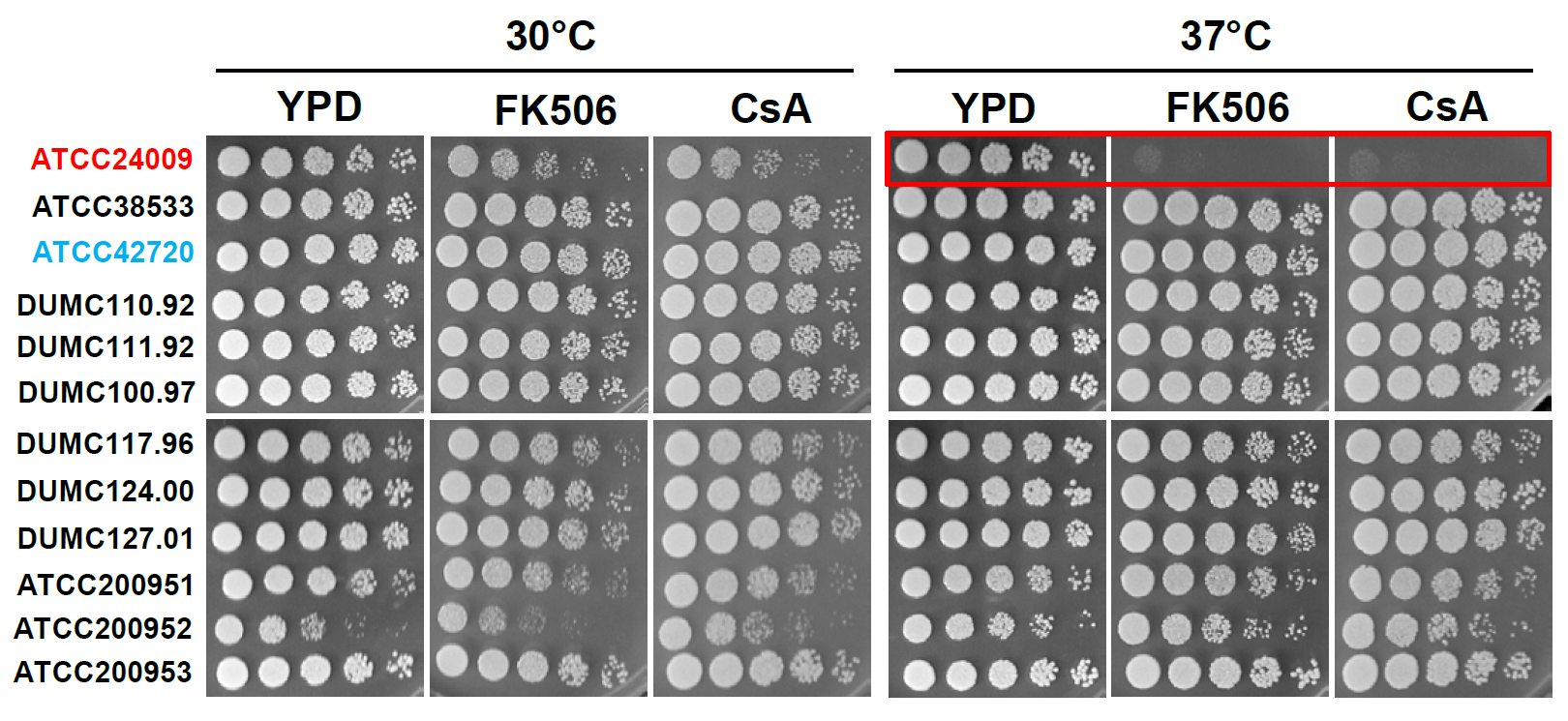

Supplement: Figure S3 — Few C. lusitaniae isolates exhibit temperature-sensitive growth when exposed to calcineurin inhibitors. (TIF) [file pone.0044192.s003.tif]

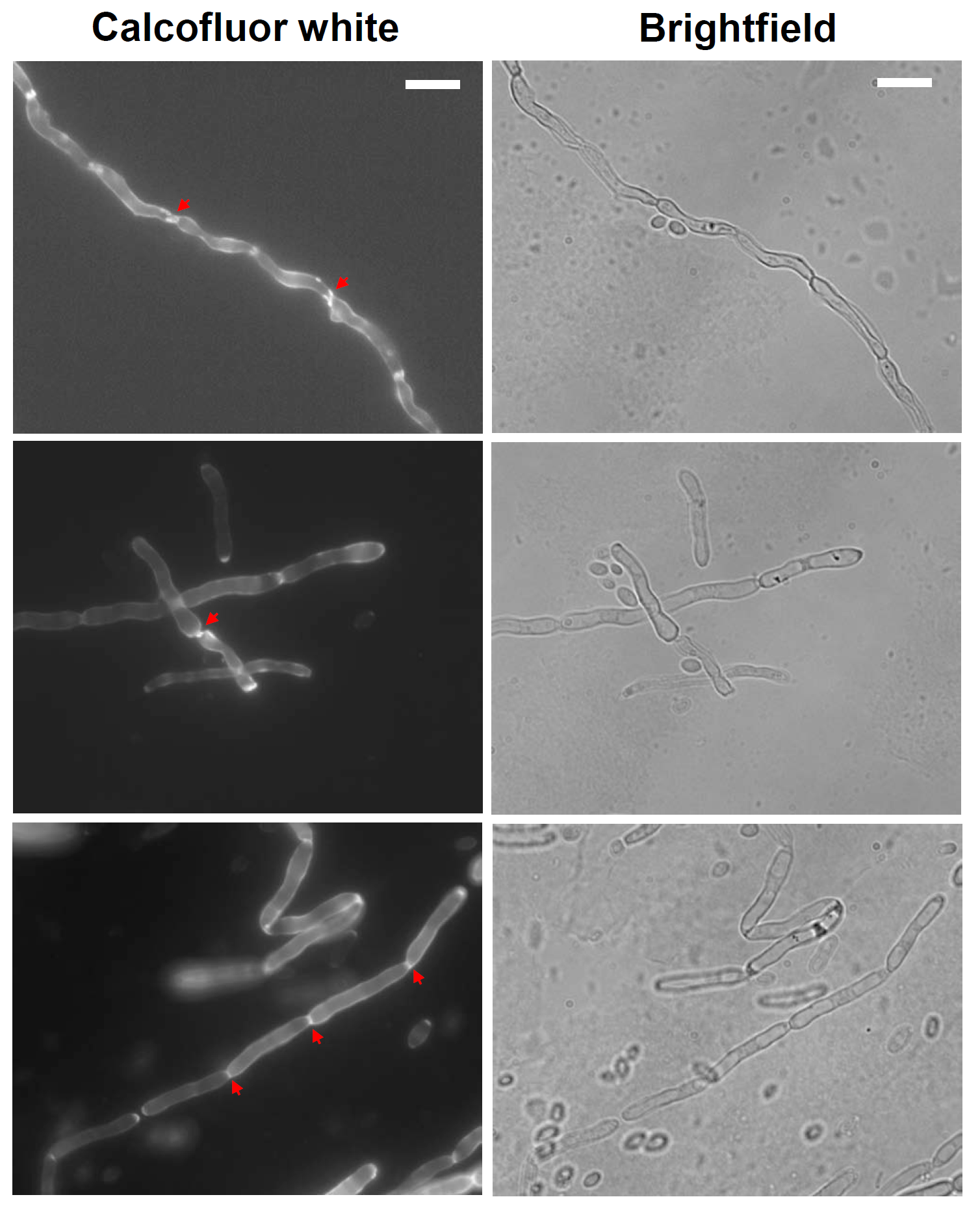

Supplement: Figure S4 — C. lusitaniae produced pseudohyphae growth on filament-inducing solid agar medium. (TIF) [file pone.0044192.s004.tif]

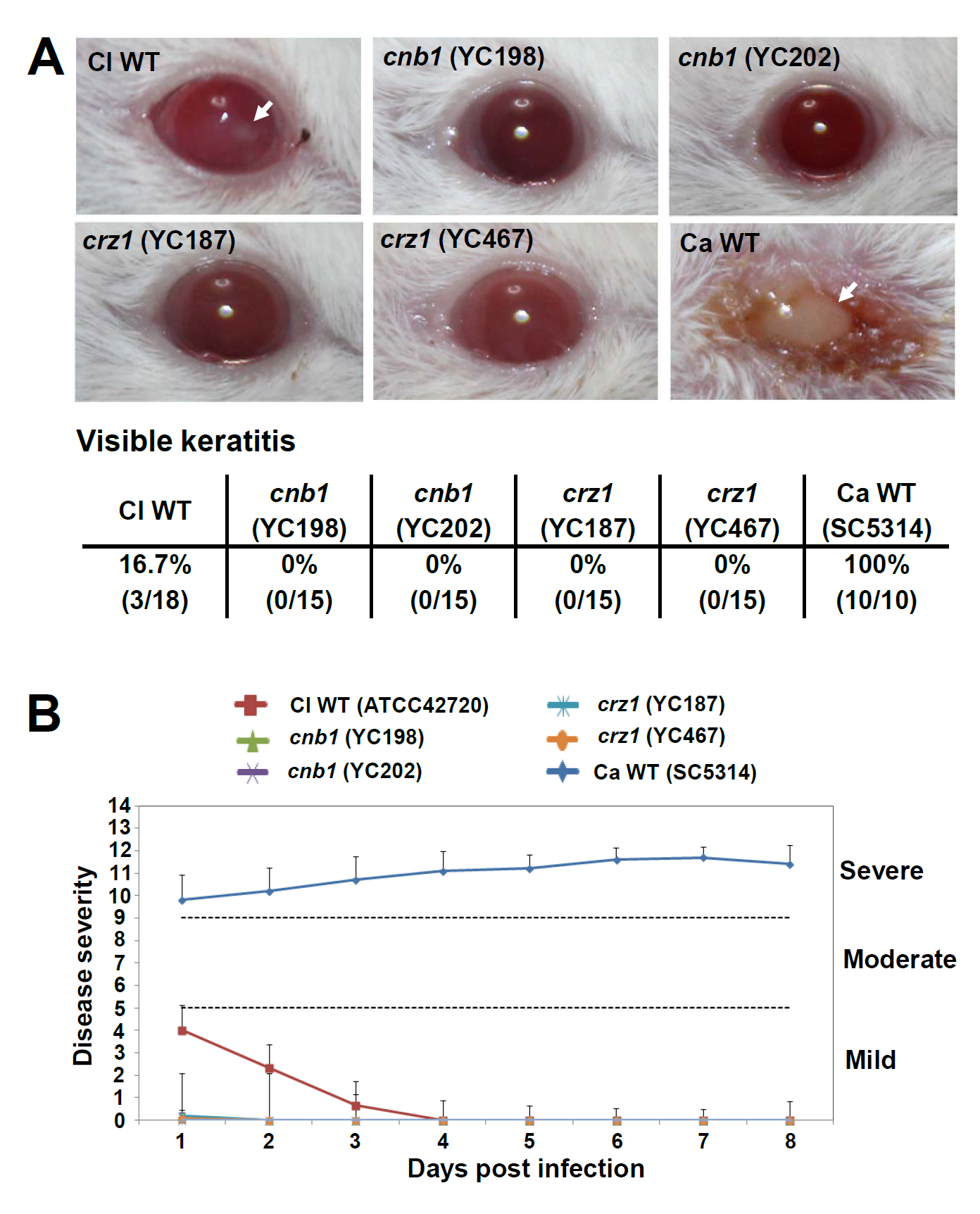

Supplement: Figure S5 — Clinical outcomes of ocular inoculations with C. lusitaniae wild-type (ATCC 42720), cnb1 mutants (YC198 and YC202), crz1 mutants (YC187 and YC467), or C. albicans wild-type (SC5314). (TIF) [file pone.0044192.s005.tif]
